# Real-World Pattern of Treatment and Clinical Outcomes of EGFR-Mutant Non-Small Cell Lung Cancer in a Single Academic Centre in Quebec

**DOI:** 10.3390/curroncol28060434

**Published:** 2021-12-07

**Authors:** Jason S. Agulnik, Goulnar Kasymjanova, Carmela Pepe, Manjusha Hurry, Ryan N. Walton, Lama Sakr, Victor Cohen, David Small

**Affiliations:** 1 Peter Brojde Lung Cancer Centre, Jewish General Hospital, McGill University, Montreal, QC H3T 1E2, Canada; jagulnik@jgh.mcgill.ca (J.S.A.); cpepe@jgh.mcgill.ca (C.P.); lsakr@jgh.mcgill.ca (L.S.); vcohen@jgh.mcgill.ca (V.C.); dsmall@jgh.mcgill.ca (D.S.); 2 AstraZeneca Canada, Mississauga, ON L4Y 1M4, Canada; manjusha.hurry@astrazeneca.com (M.H.); ryan.walton@astrazeneca.com (R.N.W.)

**Keywords:** NSCLC, EGFR, TKI treatment, real-world treatment pattern

## Abstract

The discovery of EGFR tyrosine kinase inhibitors (TKI) for the treatment of EGFR mutant (EGFRm) metastatic NSCLC is regarded as a landmark in lung cancer. EGFR-TKIs have now become a standard first-line treatment for EGFRm NSCLC. The aim of this retrospective cohort study is to describe real-world patterns of treatment and treatment outcomes in patients with EGFRm metastatic NSCLC who received EGFR-TKI therapy outside of clinical trials. One hundred and seventy EGFRm metastatic NSCLC patients were diagnosed and initiated on first-line TKI therapy between 2004 and 2018 at the Peter Brojde Lung Cancer Centre in Montreal. Following progression of the disease, 137 (80%) patients discontinued first-line treatment. Moreover, 80/137 (58%) patients received second-line treatment, which included: EGFR-TKIs, platinum-based, or single-agent chemotherapy. At the time of progression on first-line treatment, 73 patients were tested for the T790M mutation. Moreover, 30/73 (41%) patients were found to be positive for the T790M mutation; 62/80 patients progressed to second-line treatment and 20/62 were started on third-line treatment. The median duration of treatment was 11.5 (95% CI; 9.62–13.44) months for first-line treatment, and 4.4 (95% CI: 1.47–7.39) months for second-line treatment. Median OS from the time of diagnosis of metastatic disease was 23.5 months (95% CI: 16.9–30.1) and median OS from the initiation of EGFR-TKI was 20.6 months (95% CI: 13.5–27.6). We identified that ECOG PS ≤ 2, presence of exon 19 deletion mutation, and absence of brain metastases were associated with better OS. A significant OS benefit was observed in patients treated with osimertinib in second-line treatment compared to those who never received osimertinib. Overall, our retrospective observational study suggests that treatment outcomes in EGFRm NSCLC in real-world practice, such as OS and PFS, reflect the result of RCTs. However, given the few observational studies on real-world treatment patterns of EGFR-mutant NSCLC, this study is important for understanding the potential impact of EGFR-TKIs on survival outside of clinical trials. Further real-world studies are needed to characterize patient outcomes for emerging therapies, including first-line osimertinib use and combination of osimertinib with chemotherapy and potential future combination of osimertinib and novel anticancer drug, outside of a clinical trial setting.

## 1. Introduction

Lung cancer is the most common cancer worldwide, accounting for 2.1 million new cases and 1.8 million deaths in 2018 [1]. In Canada, in 2020, an estimated 21,200 people died from lung cancer. This represents 25% of all cancer deaths [2]. The treatment of lung cancer is the fastest developing area and the guidelines are continuously evolving, compared to other cancers [3]. A major advancement in the treatment of non-small cell lung cancer (NSCLC) has been the discovery of genomic abnormalities (or oncogenic drivers) that drive the development of certain types of lung cancer [4,5,6]. To date, several driver mutations have been identified with the most common being GTPase (guanosine triphosphate) KRAS (Kirsten rat sarcoma viral oncogene homolog), epidermal growth factor receptor (EGFR), anaplastic lymphoma kinase (ALK), and human epidermal growth factor 2 (HER2) mutations [7]. In Western countries, EGFR mutations are present in 10–35% of NSCLC cases, but the prevalence of these mutations is not uniform among populations; it tends to be more prevalent in females, non-smokers, and patients with bronchioloalveolar features in tumor specimens [8,9,10]. 

The discovery of EGFR tyrosine kinase inhibitors (TKI) for the treatment of EGFRm metastatic NSCLC was regarded as a landmark in lung cancer. The economic model, where a new intervention is compared to the current standard of treatment, is primarily based on randomized clinical trials (RCT). RCT evidence leads to regulatory approval and incorporation of novel treatments into practice. It is well known from RCTs that first and second-generation EGFR-TKI therapies are superior to chemotherapy in EGFR-mutant (EGFRm) metastatic lung cancer patients [8,11,12,13,14,15,16,17]. As a result, EGFR-TKIs have now become a standard first-line treatment for EGFRm NSCLC [18,19]. Recently, osimertinib, a third-generation EGFR-TKI has been approved in multiple countries, including Canada, for the treatment of patients with locally advanced or metastatic EGFRm NSCLC in the first setting based on the superiority of osimertinib compared to first-generation EGFR-TKI [20].

However, there is little published observational data on the survival of EGFRm NSCLC patients treated with EGFR-TKI outside of RCTs [21,22,23]. In particular, some of the real-world data that has yet to be published includes population characteristics at diagnosis of EGFRm NSCLC, treatment types, and associated survival outcomes. 

The aim of this retrospective cohort study is to describe real-world patterns of treatment and treatment outcomes in patients with EGFRm metastatic NSCLC who received EGFR-TKI therapy outside of clinical trials. To our knowledge, this is the first study reporting the trajectory of treatment and outcomes of EGFRm NSCLC patients in the province of Quebec. Although the results of this analysis will never replace RCTs, they do offer complementary information for physicians, patients, and policymakers. 

## 2. Materials and Methods

### 2.1. Objectives

This is a retrospective observational cohort study of an EGFRm metastatic NSCLC patient population which aims to assess real-world clinical treatment patterns, and treatment outcomes.

Primary objective:

- To describe the treatment patterns and outcomes including real-world progression-free survival (rwPFS), response rate (RR) and overall survival (OS) for EGFRm NSCLC patients treated at an academic centre in Montreal, Canada

Secondary objective:

- To describe demographic and clinical characteristics at diagnosis of EGFRm NSCLC patients and their prognostic value

### 2.2. Study Design

This is an observational retrospective cohort study of patients diagnosed with metastatic EGFRm NSCLC at the Peter Brojde Lung Cancer Centre in Montreal, Quebec. Data were extracted from the lung cancer registry. The cancer registry contains data on all lung cancer cases treated at our center since 2001. As a routine practice, every patient diagnosed with lung cancer is entered into the registry in real time. The information is updated as treatment changes. The study was approved by the Jewish General Hospital Research Ethics Committee (Protocol: JGH-07-025). 

### 2.3. Patients Selection Criteria

For purpose of this study, all the cases treated between 2004 and 2018 were screened for inclusion: eligible patients had to be initiated on first-line EGFR-TKI treatment for metastatic EGFRm NSCLC. 

We extracted the following information:

- Patient characteristics including date of metastatic NSCLC diagnosis, stage at the time of initial diagnosis, sex, age at the time of diagnosis, ethnicity, Eastern Cooperative Oncology Group performance status (ECOG PS), smoking history.
- Tumor characteristics include the stage of the disease, presence or absence of brain metastases, and type of EGFR mutations.
- Type of molecular tests: the evolution of the EGFR test for our center is recorded as: Denaturing high-pressure liquid chromatography (DHPLC) from 2004 to 2007; single gene sequencing from 2008 to 2010; real-time polymerase chain reaction (PCR) from 2011 to 2014 and next-generation sequencing (NGS) from 2014 to current.
- Real-world treatment patterns identified in our clinical practice include comprehensive treatment history from diagnosis to current treatment, treatment duration, and modality sequencing (targeted therapy, chemotherapy, radiotherapy, and best supportive care).

Patients who were initiated with systemic chemotherapy prior to EGFR results and subsequently switched to EGFR-TKI prior to disease progression were also included in the analysis. To ensure the sufficient maturity of survival data and the homogeneity of treatment, only patients diagnosed with metastatic NSCLC from September 2004 to December 2018 were used for the study. The database was locked for analysis on 31 May 2019. Patients referred to our center for a second opinion were excluded due to a lack of follow-up information.

### 2.4. Outcome Measures and Definitions

The index date was set to date of diagnosis of metastatic NSCLC.

- Treatment patterns are described by line of treatment. For the purpose of this study, first-line therapy was defined as EGFR-TKI treatment commencing after the index date.
- Duration of EGFR-TKI treatment was calculated as the time (in months) elapsed between the start and end dates of the treatment.
- Response to treatment was defined by the treating physician as per RECIST criteria and was based on radiographic imaging (CT/PET) and categorized for analysis purposes as an objective response (complete response (CR) + partial response (PR), stable disease (SD) and progressive disease (PD).
- rwPFS was defined as the time between initiation of EGFR-TKI and clinician-defined progression based on RECIST [24]. In patients who were initiated with chemotherapy, but subsequently were tested positive for EGFR mutation, disease progression was calculated from the start of EGFR-TKI treatment to the date of progression on this treatment.
- OS is defined as the time from index date to the date of death or last follow-up. Patients were censored if they were lost to follow-up or the event (death) did not occur within the study duration.

### 2.5. Statistical Analysis

Demographics, clinical characteristics, and treatment patterns are described using frequencies and proportions for categorical data and using means with standard deviation or medians with the associated 95% confidence interval (CI) for numeric data. Time variables (OS, rwPFS) are reported as medians with 95% CI using Kaplan–Meier statistics. 

To avoid immortal time bias, the landmark survival method was used for osimertinib survival analysis. Landmark time was set as a start date of second-line therapy. Only events that occurred during that period were counted for this analysis. 

Cox regression analysis was performed to identify prognostic factors for survival. The following factors were included in the model: sex, race, smoking, ECOG PS, type of EGFR mutation, type of EGFR-TKI testing. 

Statistical analyses were conducted using IBM SPSS statistics 20 software for Windows.

## 3. Results

### 3.1. Patients

Between September 2004 and Dec 2018, 1229 patients were diagnosed with metastatic NSCLC patients. Of those 170 (12%) patients were EGFRm and started on first-line EGFR-TKI treatment. 

Table 1 summarizes the characteristics of the study cohort. The majority of patients were female (71%), Caucasian (69%) and life-long non-smokers (62%); the mean (SD) age was 65 (12) years. Forty-two patients (25%) were initially diagnosed with early/locally advanced EGFRm NSCLC and later progressed to the metastatic stage: 31 of them had surgery and 11 received curative-intent radiation. The most common molecular alterations were exon 19 deletion (58%) and exon 21 (L858R) (37%) mutations. Fifty-one patients presented with brain metastasis at the time of diagnosis and received brain radiation (WBRT or SRS). 

### 3.2. First Line EGFR-TKI Treatment at the Time of Diagnosis of Advanced Disease

**Figure 1:**
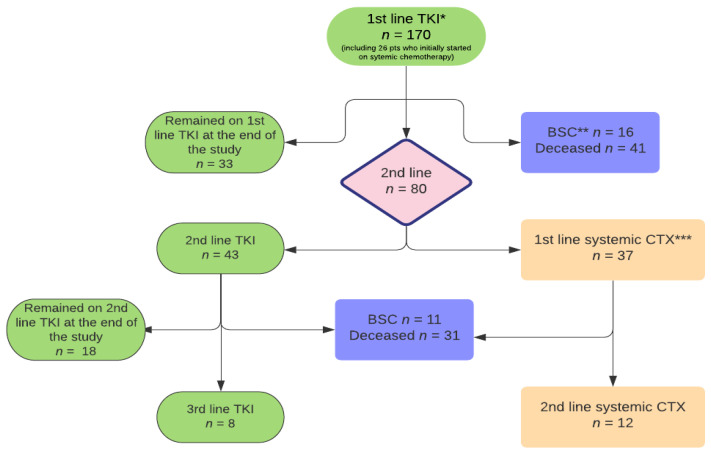
Flow chart of treatment. * TKI—tyrosine kinase inhibitors; ** BSC—best supportive care; *** CTX—chemotherapy.

All 170 patients received EGFR-TKI treatment for metastatic EGFRm NSCLC (Figure 1). Twenty-six patients (15%) diagnosed between 2004 and 2009 initially received a median of three cycles of systemic therapy prior to EGFR-TKI due to a delay in EGFR testing results. The EGFR-TKI treatment was initiated before the disease progression, and they were included in the analysis.

Of the 170 patients, 110 (65%) patients received gefitinib, 56 (33%) received erlotinib and 4 (2%) received afatinib (Table 2). The median duration of first-line EGFR-TKIs treatment was 11.5 (95% CI; 9.62–13.44) months; the range was 0.5 to 98 months. There was no significant difference in treatment duration among the types of EGFR-TKI used. 

Table 3 summarizes the first-line treatment outcomes. Following progression of the disease, 137/170 (81%) patients discontinued the first-line TKI treatment and 33/170 (19%) patients were still on the first-line TKI at the end of the study (30 May 2019). Out of 137 patients who discontinued first-line TKI treatment, 57 (42%) patients did not receive any further treatment: 41 patients died, and 16 patients received best supportive care. Eighty (58%) patients received second-line treatment.

### 3.3. Second Line Treatment for Advanced Disease

The second-line treatment included: EGFR-TKIs, platinum-based chemotherapy or single-agent chemotherapy (Figure 1 and Table 2). Out of 80 patients who progressed and received second-line treatment, 43 (54%) patients received next-generation EGFR-TKIs and 37 (46%) were treated with systemic chemotherapy (Table 2). Among patients treated with systemic chemotherapy, 16/37 (43%) received platinum-based regimens and 21/37 (57%) received single-agent chemotherapy (Table 2). At the end of the study, 18 patients remained on second-line TKI. Of those, 15 were on osimertinib, 2 on afatinib and 1 on gefitinib. 

T790M resistance mutation testing became available in 2015 and the presence of T790M resistance mutation was required for osimertinib treatment until January 2018. At the time of progression on first-line treatment, 73 patients were tested for the T790M mutation; 30/73 (41%) patients were found to be positive for T790M. In 12/30 (40%) of these cases, the T790M mutation was found on the circulating tumor DNA (ctDNA) test which became available in our center in 2017. The remaining 18 (60.0%) patients were found to be T790M positive on tissue biopsy samples. All 30 T790M patients were treated with osimertinib as their second-line treatment. 

The median duration of second-line therapy was 4.4 (95% CI: 1.47–7.39) months. The duration of osimertinib treatment was 14.8 (95% CI: 2.05–27.47) months which was significantly longer compared to other TKI regimens (Table 2). 

### 3.4. Third Line Treatment for Advanced Disease

Of the 80 patients who started second-line therapy, 62 (78%) progressed. Of those, 20 (32%) received a third-line therapy (Table 2), 31 (50%) died and 11 (18%) received BSC. Among treated patients, the majority (12/20) received systemic chemotherapy and 8/20 received other TKI therapies. 

### 3.5. rwPFS and OS Analysis

**Figure 2:**
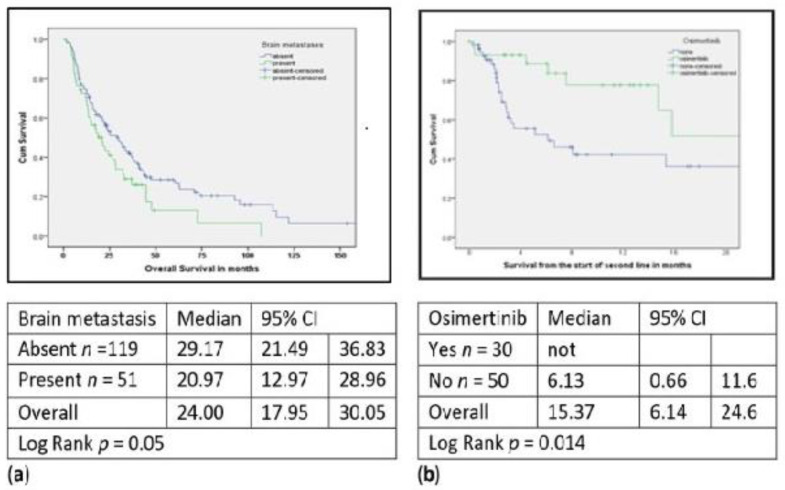
(**a**) OS in EGFRm patients with and without brain metastasis; (**b**) OS in EGFRm patients with and without osimertinib.

The median follow-up was 37 months (range 5–122); 127 death events were observed. Median OS from the time of diagnosis of metastatic disease was 23.5 months (95% CI: 16.9–30.1) and median OS from the initiation of EGFR-TKI was 20.6 months (95% CI: 13.5–27.6). In patients without brain metastases, the OS was significantly better compared to those with brain metastases: 29.5 (95% CI: 21.0–39.7) vs. 20.9 (95% CI; 12.9–28.9) months respectively, *p* = 0.05 (Figure 2a). There was no difference in OS (*p* = 0.60) in comparing 26 patients who were switched to the EGFR-TKI before disease progression to 144 patients initiated on EGFR-TKI, 23.5 months (95% CI: 16.9–30.1) and 25.1 months (95% CI: 10.1–40.10), respectively. 

A significant survival benefit was observed in patients treated with osimertinib in second-line treatment compared to those who did not receive osimertinib when landmark survival analysis was used (Figure 2b). The starting point of second-line therapy was designated as a landmark time for survival. Median survival for patients on osimertinib was not reached at 24.8 months. 

There were 170 patients available for analysis of the best response to treatment. The objective response rate of first-line EGFR-TKI therapy was 74.7%: 83/170 (49%) had CR/PR, 44/170 (26%) had SD. The progression rate was 25.3% (43/170 patients). When compared to type of TKI used in the first-line setting, gefitinib provided a significantly higher response rate (55%) and lower progression rate (19.0%) *p* = 0.005 compared to erlotinib (Table 4).

rwPFS in first-line EGFR TKI therapy was 11.2 (95% CI: 10.1–15.3) months. No difference in rwPFS among different EGFR-TKIs was observed. In the second-line treatment, PFS was 3.1 (1.8–5.9) months with significantly (*p* < 0.001) prolonged PFS in patients treated with osimertinib 14.7 (95% CI: 9.51–20.1) compared to other EGFR-TKIs 2.5 (1.88–3.18).

Several patient/tumor characteristics identified at the time of diagnosis of metastatic EGFRm NSCLC were considered to be potential prognostic factors for survival in univariate analysis: ECOG PS at index date (≤2 vs. >2), presence of brain metastases (yes vs. no), and EGFR mutation (exon 19 (E19del) vs. exon 21 (L858R). Patients with exon 19 deletion had a significantly better OS (29.1months, 95% CI: 22.9–35.3) when compared to exon 21 (16.03 months, 95% CI: 11.2–20-8). 

Multivariate Cox proportional hazards regression revealed that exon 19 (E19del) mutant patients experienced a lower risk of death (HR: 1.27; 95% CI: 1.1–2.4) compared to other mutations, with adjustment for potential confounding variables (Table 5). Several other variables were associated with increased risk of death: ECOG PS > 2 (HR: 0.45; 95% CI: 0.3–0.8) and presence of brain metastases (HR: 0.65; 95% CI: 0.4–0.9). 

## 4. Discussion

This review was performed to determine whether—in patients with EGFRm metastatic NSCLC—real-world outcomes achieved with TKI therapy are comparable to RCT outcomes reported in RCTs. Current Canadian guidelines recommend first-line EGFR-TKI monotherapy for EGFRm metastatic NSCLC patients, based on the results of superior OS, PFS and RR of first-line TKI compared to chemotherapy [18,25]. In 2018, osimertinib was approved for first-line treatment of EGFRm NSCLC and has become the new standard of care for these patients [20,26]. Osimertinib was not available for first-line treatment in our retrospective study. Despite the high efficacy of EGFR TKIs observed in metastatic EGFRm NSCLC, resistance emerges in most patients. The most common mechanism of resistance to first- and second-generation EGFR TKIs arises from the development of a secondary mutation in exon 20 (T790M) [27,28]. Our patients acquired a resistance to first- and second-generation TKIs after approximately 12 months of treatment in the first-line setting. At the time of progression, most patients were tested for the T790M resistance mutation. Forty-one percent were found to be positive and received osimertinib. The rate of the acquired T790M mutation resistance in our study was slightly lower than the 51–68% reported by Oxnard et al. [29]. The second-line treatment patterns we identified are similar to those recommended in the guidelines [18,19]: 54% of these patients were started on another TKI and 34% started on systemic chemotherapy. 

Some studies reported that osimertinib as second- or third-line TKI treatment outperforms systemic chemotherapy and first/second-generation TKIs [30,31,32]. The duration of second- or third-line treatment with osimertinib was in concordance with the AURA extension trial as well as the ASTRIS trial [30,33]. An RCT of second-line therapy with osimertinib, or cisplatin and pemetrexed, in NSCLC patients with T790M mutation, demonstrated superior efficacy for second-line osimertinib [31]. 

Real-world data reported from other Canadian provincial institutions demonstrated similar outcomes in terms of OS and PFS. However, the proportion of patients receiving second- or third-line treatment varied between institutions depending on local clinical practice [22,34]. 

Survival of 20.6 months from the time of TKI initiation in our study was comparable to 19–27 months from RCT results [32,35,36] as well as to 17.6–25 months from other real-world settings [23,34,37,38]. In our cohort, the OS in patients with brain metastasis was 21 months, which was similar to 22 months reported by Fujita et al. [39]. 

The response rate and PFS in the first-line TKI (gefitinib) were in concordance with the LUX Lung-7 trial [40]. Although it was reported by Ezeife et al. that afatinib use in pretreated patients prolongs survival by 5 months, it was not routinely used in our practice [41]. The significant prognostic factors for survival in our cohort were not different from those reported from the majority of the randomized controlled phase III trials [11,36,42] and in real-world studies [12,22,43]. Patients with good performance status, exon 19 deletion mutation and without brain metastases were found to have prolonged survival in our study. In addition, the method used for EGFR testing has also been found to be a prognostic factor. This could be explained by advances in technology of the testing methods. 

This is a retrospective single-center study. While the registry provided accurate information on demographic characteristics, treatment patterns and survival data, as with all retrospective studies, there may be unmeasured confounding factors or unidentified sources of bias. Moreover, the number of patients treated with afatinib is relatively small, as only four patients received this treatment. In addition, the data were collected prior to the approval of osimertinib for first-line treatment and before the widespread use of immunotherapy treatments for metastatic NSCLC treatment.

## 5. Conclusions

Overall, our retrospective observational study suggests that treatment outcomes in EGFRm NSCLC in real-world practice, such as OS and PFS, reflect the result of RCTs. However, given the few observational studies on real-world treatment patterns of EGFR-mutant NSCLC, this study is important for understanding the potential impact of EGFR-TKIs on survival outside of clinical trials. We identified that ECOG PS ≤ 2, presence of Exon 19 deletion mutation, and absence of brain metastases were associated with better OS. Further real-world studies are needed to characterize patient outcomes for emerging therapies, including first-line osimertinib use and combination of osimertinib with chemotherapy and potential future combination of osimertinib and novel anticancer drugs, outside of the clinical trial setting. 

## Figures and Tables

**Table 1:** Baseline demographic and clinical characteristics of patients.

Characteristics	*N* = 170 *N* (%)
Age at diagnosis (y), interquartile range (IQR)	65 (IQR: 18.25)
Sex	
Female	121 (71)
Male	49 (29)
Race	
Caucasian	117 (69)
Asian	45 (27)
Black	8 (4)
Smoking	
Never-smoker	106 (62)
Ever-smoker	64 (38)
ECOG PS	
0–1	151 (89)
2	19 (11)
>2	0
Stage at Diagnosis	
Early stage	23 (14)
Locally advanced	19 (11)
Metastatic	128 (75)
EGFR mutation	
Exon 19 (E19 del)	98 (58)
Exon 21 (L858R)	64 (37)
Exon 18 (E181) ^1^	7 (4)
Exon 20 insertion (E20)	1 (1)
Type of molecular test:	
DhPLC (2004–2007)	32 (19)
Single gene sequencing (2008–2010)	17 (10)
Real-time PCR (2011–2014)	48 (28)
NGS (2015-current)	73 (43)
Brain metastases at diagnosis	
Present	51 (30)
Not present	119 (70)
Prior therapies for advanced/metastatic NSCLC before TKI	
Yes	26 (15)
No	144 (85)

^1^—includes: p.Glu709Ala; p.Glu709_Thr710delinsAsp; p.Gly719Ala.

**Table 2:** Lines EGFR-TKI treatment.

EGFR-TKI	Frequency *N* (%)	Duration of Treatment Median (95% CI) in Mo	*p*-Value
**First Line**			
Gefitinib	110 (64.7)	11.7 (8.03–15.36)	0.535
Erlotinib	56 (32.9)	11.4 (7.4–15.29)	
Afatinib	4 (2.4)	not reached	
Total	170 (100)	11.5 (9.62–13.44)	
**Second Line**			
Osimertinib	30 (37.5)	14.8 (2.05–27.47)	0.001
Erlotinib/Gefitinib	5(6.3)	2.1 (0.45–3.89)	
Afatinib	8 (10.0)	1.9 (036–3.64	
Systemic chemo	37 (46.2)	2.5 (1.83–3.24)	
Total	80 (100)	4.4 (1.47–7.39)	
**Third Line**			
Osimertinib	2 (10.0)	All censored	n/a
Gefitinib/Erlotinib	4 (20.0)	All censored	
Afatinib	2 (10.0)	All censored	
Systemic chemo	12 (60.0)	2.8 (1.29–5.91)	
Total	20 (100)	3.9 (0.74–6.46)	

**Table 3:** Outcomes of first-line treatment.

First-Line Outcomes	*n* (%)	Reason for Discontinuation *n* (%)	
Continued on First line	33 (19)		
Discontinued	137 (81)	Started second line	80 (58)
		BSC *	16 (12)
		Died	41 (30)
Total *n* (%)	170 (100)		137 (100)

* BSC—best supportive care.

**Table 4:** Response rate to first-line EGFR-TKI.

Response Rate (RR)	Afatinib	Gefitinib	Erlotinib	Total
CR +PR *n* (%)	4 (100%)	61 (55.4%)	18 (32.1%)	83 (48.8%)
SD *n* (%)	0	28 (25.4%)	16 (28.6%)	44 (25.8%)
PD *n* (%)	0	21 (19.0%)	22 (39.3%)	43 (25.4%)
Total *n* (%)	4 (100%)	110 (100%)	56 (100%)	170 (100%)

CR—complete response; PR—partial response; SD—stable disease; PD—progressive disease. *p* = 0.005.

**Table 5:** Cox regression analysis for OS from the time of initiation of EGFR-TKI.

Variable	Comparator	Cox Regression Analysis					
		Univariate Analysis		*p*-Value	Multivariate Analysis		*p*-Value
		HR	95%CI		HR	95% CI	
Female	Male	0.91	0.62–1.3	0.65	0.91	0.6–1.4	0.66
Never-smoker	Ever-smoker	0.83	0.58–1.2	0.33	1.29	0.8–1.9	0.21
ECOG PS > 2	≤2	0.44	0.3–0.8	0.005	0.45	0.3–0.8	0.004
Exon 21/20/18	Exon 19 *	1.39	1.0–1.9	0.03	1.27	1.1–2.4	0.05
Gefitinib **	Erlotinib	0.83	0.5–1.2	0.31	1.10	0.7–1.7	0.67
Brain metastasis present	Absent	1.50	1.0–2.2	0.05	1.50	1.1–2.3	0.04
Non-Asian	Asian	1.26	0.8–1.9	0.28	1.21	0.8–1.9	0.43
NGS	Alternate test type ***	2.07	1.4–3.0	<0.001	2.25	1.4–3.5	<0.001

* For the purpose of this analysis, Exon 19 mutation compared to all the other. ** Four patients treated with Afatinib excluded from these analyses. *** NGS compared to combined previous testing technic.

## Data Availability

The data presented in this study are available on request from the corresponding author.
